# Human iPSC-based Cardiac Microphysiological System For Drug Screening Applications

**DOI:** 10.1038/srep08883

**Published:** 2015-03-09

**Authors:** Anurag Mathur, Peter Loskill, Kaifeng Shao, Nathaniel Huebsch, SoonGweon Hong, Sivan G. Marcus, Natalie Marks, Mohammad Mandegar, Bruce R. Conklin, Luke P. Lee, Kevin E. Healy

**Affiliations:** 1 Department of Bioengineering and California Institute for Quantitative Biosciences (QB3), University of California at Berkeley, Berkeley, California 94720, USA; 2 Department of Materials Science and Engineering, University of California at Berkeley, Berkeley, California 94720, USA; 3 Department of Electrical Engineering and Computer Science, University of California at Berkeley, Berkeley, California 94720, USA; 4 Gladstone Institute of Cardiovascular Disease, San Francisco, California 94158, USA; 5 Department of Medicine, Division of Genomic Medicine, UCSF, San Francisco, California 94143, USA

## Abstract

Drug discovery and development are hampered by high failure rates attributed to the reliance on non-human animal models employed during safety and efficacy testing. A fundamental problem in this inefficient process is that non-human animal models cannot adequately represent human biology. Thus, there is an urgent need for high-content *in vitro* systems that can better predict drug-induced toxicity. Systems that predict cardiotoxicity are of uppermost significance, as approximately one third of safety-based pharmaceutical withdrawals are due to cardiotoxicty. Here, we present a cardiac microphysiological system (MPS) with the attributes required for an ideal *in vitro* system to predict cardiotoxicity: i) cells with a human genetic background; ii) physiologically relevant tissue structure (e.g. aligned cells); iii) computationally predictable perfusion mimicking human vasculature; and, iv) multiple modes of analysis (e.g. biological, electrophysiological, and physiological). Our MPS is able to keep human induced pluripotent stem cell derived cardiac tissue viable and functional over multiple weeks. Pharmacological studies using the cardiac MPS show half maximal inhibitory/effective concentration values (IC_50_/EC_50_) that are more consistent with the data on tissue scale references compared to cellular scale studies. We anticipate the widespread adoption of MPSs for drug screening and disease modeling.

The current drug development process is inefficient and expensive. The average time to develop and launch a new drug is 10–15 years, and the average cost is approximately 5 billion USD^1^,^2^. Preclinical development and clinical studies account for 63% and 32% of this cost, respectively^3^. A significant limitation in the restricted predictability of preclinical screening is that it relies heavily on animal models and animal derived cell lines. Due to inter-species differences in ion channels, biological pathways, and pharmacokinetic properties^4^ animal models fail to fully recapitulate human physiology, and thus in many cases do not faithfully predict human cardiotoxicity. Simple high throughput *in vitro* assays play a crucial role in assessing the safety of cardiac drugs by monitoring, for instance, currents through ‘hERG’ ion channels due to their role in causing life-threating arrhythmias^5^. Yet, the heavy reliance on simplified model cell lines (e.g., HEK293, CHO) overexpressing hERG ion channels is risky. These assays produce false positives (Verapamil) and false negatives (Alfuzosin)^6^, putting human lives at risk and resulting in a waste of valuable resources during clinical drug development.

To mimic the anisotropic structure of the cardiac ventricles, Grosberg *et al.*^7^ employed mouse embryonic stem cell derived cardiomyocytes (CMs) to make a 2D anisotropic cardiac muscle tissue by microfabrication of fibronectin layers. While they successfully showed that stimulated CMs cultured in serum aligned in the 2D structure and generated contractile force, the model is not scalable to generate a 3D thick volume tissue. Using the same cell line but a different approach, Boudou *et al.*^8^ created freestanding 3D tissue constructs and examined the effect of various drugs. Besides issues arising from the lack of biomimicry, the applicability of both systems for drug testing are limited due to the use of animal cells. With the discovery of patient-specific human induced pluripotent stem cells (hiPSCs)^9^,^10^, it is now possible to engineer human *in vitro* systems amenable for drug screening. Advances in generating stem cell derived human cardiomyocytes offer an unprecedented opportunity to create systems, which are better predictors of drug toxicity. In recent years, considerable progress has been made on the use of static 2D dish culture with human stem cell derived CMs^11^,^12^,^13^,^14^. However, the predictability of such systems is still limited, as they do not recapitulate the human *in vivo* structure and organization of the myocardium. To address this other groups introduced multiple types of 3D tissue constructs based on different methods and cell types (hESC, hiPSCs)^8^,^15^,^16^,^17^,^18^. However, all of them are restricted to the use of a static bath to deliver the nutrients and drugs, hence do not provide microcirculation like the vasculature, which includes the capability to continuously exchange the nutrients, constant exposure of the tissue to fresh drug in the plasma, and the convective-diffusive oxygen/nutrient transport.

We hypothesized that combining the genetic background of human cells with appropriate biophysical tissue architecture and “tissue-like” drug gradients would recapitulate a minimal organoid of the human myocardium sufficiently to allow accurate prediction of the cardiotoxicity of drugs. To test this hypothesis, we have developed a microphysiological system (MPS) that: i) uses 3D confinement with biomimetic dimensions to facilitate a self-organization of hiPSC derived cardiomyocytes into an aligned 3D μ-tissue; and, ii) mimics the shear flow protection of the endothelial barrier. We applied this system to test the cardiac response with four model drugs. Our results suggest that this hiPSC-derived cardiac-MPS could significantly improve the ability to prognosticate on drug efficacy and toxicity *in vitro*, reducing both the cost and duration of bringing a new drug candidate to market.

## Results

**Figure 1:**
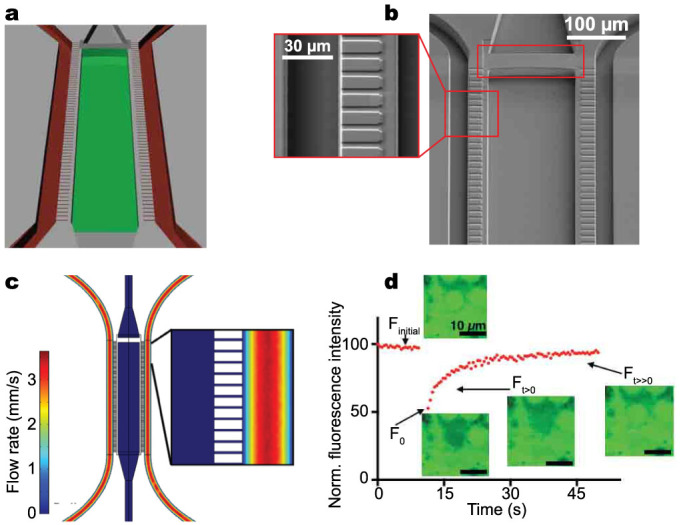
The cardiac microphysiological system (MPS). (a) Schematic of the MPS nutrient channels (red) and cell-loading channel (green). (b) Scanning electron micrograph of the MPS showing in inset the 2 μm endothelial-like barriers connecting the nutrient channel and the cell channel. Red rectangular box shows the weir that enables efficient and consistent loading of singularized high density hiPSC-CMs. (c) Schematic of the cardiac MPS showing nutrient inlet and outlet ports connected to tubes. (d) Simulated velocity profile of flow in the MPS, inset shows the magnified view. Note the lack of convection within the diffusive barriers and predominant convective flow through the nutrient channels. Thus, mass transport to the tissue is exclusively diffusive. (e) Diffusion dynamics in the microphysiological system. Normalized fluorescence recovery of 4 kDa FITC–dextran (0.2 mg/mL). Insets are confocal microscopy images corresponding to the FRAP experiment. F_initial_ is the time regime that corresponds to the initial fluorescence before bleaching; F_0_ is the fluorescence measurement immediately after photobleaching; F_t > 0_ corresponds to the recovery of fluorescence after photobleaching; F_t ≫ 0_ corresponds to maximal recovery of fluorescence at the end of the experiment. Scale bar: 10 μm.

To mimic a minimal organoid structure of the heart we have developed a cardiac microphysiological system (MPS) (Fig. 1 a–b) based on three major concepts: a) aligned three-dimensional tissue structure, inspired by the human myocardium by recapitulating the geometry of the perimysial collagen fiber spacing in the human heart (width 100–200 μm)^19^; b) microcirculation mimicking the *in vivo* transport, which includes continuous exchange of nutrients, constant exposure of the tissue to fresh drug compounds, and removal of metabolic waste products; and, c) shear flow protection of the tissue and controlled diffusive transport to the tissue.

The major components of the device are a central cell chamber, two adjacent media channels, and arrays of connecting microchannels. The media channels (width 30–40 μm) recapitulate the vasculature and allow for a precise and computationally predictable delivery of nutrients and drugs in microfluidic perfusion chambers. The cell chamber is connected to the media channels by 2 μm wide microchannels, which mimic endothelial barriers separating the cardiac microtissue from perfusion medium, protecting the tissue itself from shear forces, but allowing nutrient delivery via diffusion from the ‘vascular’ channels. The narrow cross-section of these microchannels creates a total fluidic resistance into the cell culture area 10 times greater than through the media channel, and a convection/diffusion ratio (Peclet number) of 693 in the flow channel and ≈0 in the barrier channels. Thus, the transport from the media channels to the cell chamber is purely diffusive (Fig. 1c), featuring diffusion properties characteristic of the endothelial barrier for humans *in vivo*. The diffusive transport was verified by performing fluorescence recovery after photobleaching (FRAP) experiments on the MPS (Supplementary Movie 1), yielding a characteristic diffusion time of 2.5 s for 4 kDa FITC-dextran (Fig. 1d).

To create a robust and reproducible hiPSC derived cardiac microtissue in the cell chamber, we relied on a consistent, well-characterized, high yield, and high-efficiency cardiomyocyte differentiation protocol (Supplementary Fig. 1). This was achieved by modifying a hiPSC-CM differentiation protocol introduced by Lian *et al.*^20^ Using optimized procedures for cell singularization and device loading, hiPSC derived CMs (hiPSC-CMs) on days 15–20 of the CM differentiation were introduced into the MPS at a high packing density while keeping pressure and stresses low (Supplementary Movie 2). Cells were loaded at this stage to achieve robust CM commitment without excessive buildup of extracellular matrix (ECM). Additionally, this early injection time point promoted the formation the cardiac tissue without use of either exogenous ECM or synthetic matrices.

**Figure 2:**
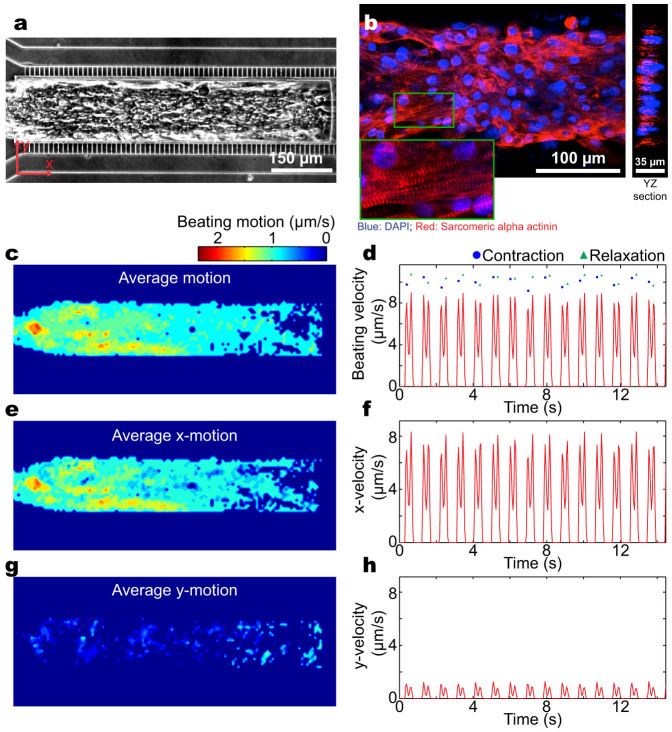
Characterization of the 3D cardiac tissue in the microphysiological system (MPS). (a) Optical microscopy image of a cardiac tissue in the MPS. (b) Confocal fluorescence microscopy imaging of the microtissue in the MPS reveals an aligned multiple cell layer thick structure. Inset shows a magnified view of the sarcomeric alpha actinin and DAPI staining. (c, d) Heat map of the time-averaged beating motion and corresponding average beating kinetics in the MPS, (e, f) along the channel axis, and (g, h) perpendicular to the channel axis.

**Figure 3:**
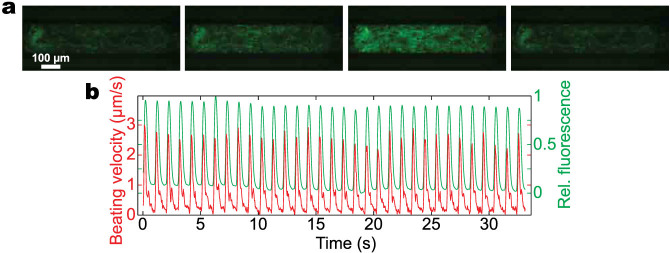
Cardiac tissue derived from a genetically engineered hiPS cell line expressing a GCaMP6 reporter in the MPS. (a) Frames from a fluorescence movie (GFP channel) showing the switching from dim to bright during activity of Ca^2+^ channels. (b) Time-course of the normalized fluorescence intensity (green) and the beating motion (red) obtained by computational analysis of the movie. This combination allows high throughput analysis of mechanical and electrophysiological properties.

The hiPSC-CMs formed a 3D cardiac tissue in serum-free media within 24 hours of loading, which started to beat spontaneously with robust and homogenous beating at physiological beat rates of 55–80 bpm without any external stimulation (Fig. 2a, Supplementary Movie 3). Within 7 days of formation, the beating became aligned in an uniaxial manner and consisted of multiple cell layers, as confirmed by confocal fluorescence microscopy (Fig. 2b). Computational motion-tracking of the beating velocity indicated the beating was aligned along the long axis of the MPS (Fig. 2 c–h). Aligned 3D cell structure and consistent physiological beating are crucial prerequisites for a cardiac MPS, since functionality is dictated by structural alignment of the cells. Drug response, for instance, is strongly dependent on the initial spontaneous beat rate of the tissue^21^ and the electromechanical activity of the heart is governed by the intracellular connections and 3D alignment of the cardiac tissue^22^,^23^. The MPS system is highly scalable and presents the opportunity to model human genetic disease via the use of patient-derived or genome-edited hiPSC-CMs in a rapid and cost effective manner. For example, we used hiPSC-CMs derived from a genetically engineered hiPSC line expressing a GCaMP6 reporter to monitor Ca^2+^ currents^21^, which provides the ability to collect electrophysiological data on a high-content basis using simple optical microscopy, as shown in Fig. 3 and Supplementary Movie 4.

To validate the drug response of the cardiac MPS we tested two pharmacological agents, Isoproterenol (β-adrenergic agonist) and E-4031 (hERG blocker), and two clinically used drugs with known clinical effects, Verapamil (multi-ion channel blocker) and Metoprolol (β-adrenergic antagonist) (Table 1). We chose these compounds since they represent four different important classes of pharmacological agents with well-characterized clinical responses. To characterize drug responsiveness, we used beat-rate as a metric. Due to the controlled dimensions and the alignment of the engineered cardiac tissue formed in our MPS, we obtain consistent and spontaneous beat rates on the order of 55–80 beats per min, allowing us to make sensitive, reproducible measurements regarding how drug treatment affects beat rate. Furthermore, the pharmacological agents tested in this study have a known effect on the beat rate. Hence, mechanical motion is an important method to analyze behavior in a non-invasive manner without the use of any synthetic dyes or complex electrophysiological measurements.

**Figure 4:**
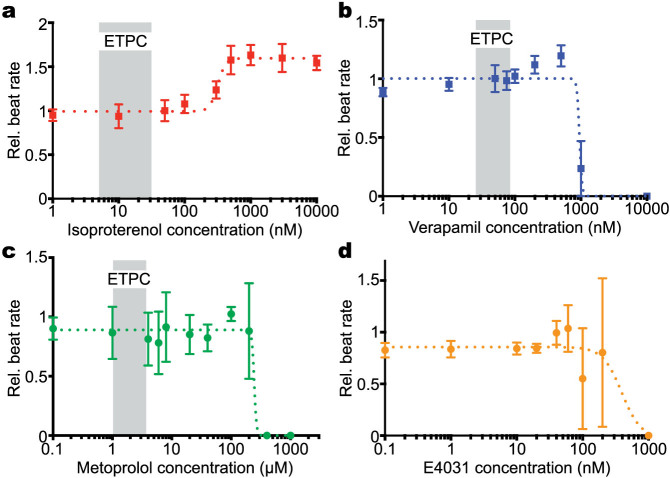
Dose-dependent study of different drugs on the cardiac MPS. (a) Isoproterenol causes a dose-dependent increase in beat rate and an EC_50_ value of 315 nM. (b) Verapamil induces a dose-dependent decrease in beat rate with incidences of arrhythmia observed at higher concentrations and an IC_50_ value of 950 nM. (c, d) Metoprolol and E4031 induce a dose-dependent decrease in beat rate with IC_50_ values of 2.3 μM and 1.9 nM respectively. Grey areas indicate unbound estimated therapeutic plasma concentration in patients (ETPC)^28^,^35^. Note there is not an ETPC highlighted for E-4031 because the drug is used solely for research purposes and only one clinical trial was conducted. Error bars indicate mean ± S.D.

Our results demonstrate good concordance with clinical observations. Administration of Isoproterenol increased the beat rate with an EC_50_ of 315 nM (Fig. 4a and Supplementary Movie 5 and 6). This value from the MPS is significantly higher than the reported values for 2D hiPSC-CMs^24^, and is comparable to contractility measurements obtained in acute experiments performed on human ventricular heart tissue slices^25^. Thus, our cardiac MPS provides data that is consistent with data obtained on adult human tissues, which are significantly limited in quantity and more likely to vary in genetic background^26^,^27^.

Verapamil is a false positive hERG blocker that has similar affinity for hERG and L-type Ca^2+^ channels. When applied to CMs, the effect on action potential duration (APD) reflects the combined block of both channels. The Ca^2+^ ion channels conduct inward current, and delayed rectifier potassium current (I_kr_) is outward, so the blocking effects are mixed and occur at different concentrations^24^,^28^. This makes animal-derived cells and their tissue models unreliable in predicting the effects of the drug. Applying various Verapamil concentrations to the cardiac MPS revealed a concentration-dependent decrease in beat rate with an IC_50_ value of 950 nM (Fig. 4b and Supplementary Movie 7 and 8). Incidences of arrhythmias at higher drug concentration and cessation of beating above 1 μM were observed. This is a significantly higher IC_50_ value compared to CMs derived from hiPSC embryoid bodies (EB) of different cellular maturity (Table 2)^24^. Most significantly, in clinical use the estimated therapeutic unbound plasma concentration (ETPC*_unbound_*) for Verapamil is 25–81 nM^28^, leading to a safety margin (IC_50_/Mean ETPC*_unbound_*) of ≈3 estimated from either 2D hiPSC-CMs experiments or ≈2–8 for EBs respectively (Table 2). These are unusually low safety margins, given that pharmaceutical companies typically target a safety margin of ≈20 to 30^28^, and therefore these assays would indicate safety issues with Verapamil, which may have prevented its clinical development. Clinical observations, however, report very few incidences of arrhythmias at the ETPC for Verapamil in patients^28^. Compared with the literature data, our MPS predicts a much higher safety margin of ≈18 and has better concordance with clinical observations and large-scale animal models (Table 2). This observation is highly significant, as we propose that in the short-term the cardiac MPS can complement animal models, and in the future may have the potential to replace animal studies, which often are expensive, unethical, and do not predict the drug's actual effect.

The physiologically relevant cardiac MPS creates human 3D aligned cardiac microtissues with drug responsiveness that is similar to the responsiveness of later-stage hiPSC-CMs. Since we use human CMs in the cardiac MPS, we alleviate issues associated with non-human ion channels. Moreover, the fluidic design delivers a precise concentration of the drug continuously during the entire drug-testing period, unlike standard 2D culture models where the drug concentration fluctuates with time and media changes. Interestingly, previous 3D models using embryoid body-derived CMs only show enhanced Verapamil resistance after prolonged (80 days) differentiation^24^. Thus, the recapitulated cardiac MPS creates human 3D aligned cardiac microtissues with drug responsiveness that is similar to the responsiveness of later-stage hiPSC-CMs.

## Discussion

The discovery of hiPSCs has added a new paradigm for drug screening, disease modeling, and personalized medicine. Generated from somatic cells, these cells are ethically unbiased as no human embryos are required to make them, the cells can be obtained in unlimited quantity, can be cultured for extended periods, and they can be differentiated into numerous cell types that are constituents of major organs. For example, cardiomyocytes derived from monolayer differentiation of hiPSCs offer the following advantages over primary CMs and EB derived CMs: 1) well characterized small molecule differentiation protocols using chemically defined serum free media yield consistent high quality CMs at a lower cost in comparison to CMs derived from EBs; 2) high efficiency differentiation protocols reduces lot-to-lot variability of CMs as well as their supporting stromal cells; 3) unlimited supply and ease of procurement as compared to native human CMs; 4) hiPSC-CMs can be obtained from patients with specific genetic disease, allowing direct clinical-to-model comparisons of disease pathophysiology to be performed; and, 5) as in primary human CMs, hiPSC-CMs express all the human ion channels thus can produce physiologically relevant systems.

While adult human CMs are clearly the gold standard against which hiPSC-CMs should be compared, logistical and ethical issues preclude the routine use of adult human CMs for drug screening. Through the use of disease-specific hiPSC-CMs, or CMs derived from hiPSCs that are engineered with specific mutations, we can gauge the ability of our model systems to measure pathophysiological differences in “wild type” cells and “disease specific cells”. The ability to recapitulate clinical phenotypes associated with adult genetic diseases *in vitro*, such as cardiomyopathy, can then provide groundbreaking metrics of how clinically relevant a given model system, including our MPS, is both in terms of measurement capability and cellular maturation.

The design and the underlying microfabrication principle of the MPS allow for massive parallelization of the system. Since the footprint of the device is on the scale of 1 mm^2^, a plate with standard multi-well plate dimension could, for instance, feature hundreds of cell culture channels and thus enable medium throughput screening. Furthermore, the combination of the MPS with the isogenic GCaMP6f reporter cell line and automatized video analysis software enables the high throughput analysis of mechanical and electrophysiological properties of the microtissue (Fig. 3, Supplementary Movie 4). The power of this approach is that the method requires no advanced infrastructure and allows for a parallelized, high-throughput analysis, which is non-invasive and very cost efficient. A further advantage of the underlying microfluidic concepts is that the cardiac MPS is easily amenable to study various structural and functional endpoints. For instance, the supernatant can be analyzed using mass spectrometry and the optical accessibility of the tissue enables the use of various live cell imaging microscopy techniques.

In summary, pharmacological studies on the cardiac MPS validate the system and show IC_50_ and EC_50_ values that are more consistent with the data on tissue-scale references compared to cellular-scale studies. We attribute this success to the design that aligns cells in the microtissue, and mimics many of the mass transport properties of the functional ventricular myocardium, such as defined tissue and fluid transport regions, and continuous nutrient exchange. Moreover, the cardiac microtissue in the MPS is cultured in a serum free medium, as the use of serum in culture medium may confound results^29^. All these attributes are important for a reliable *in vitro* drug-screening assay. Thus, compared to classical 2D systems, our data suggests that the MPS is a better predictor of clinical cardiology due to its bioinspired characteristics, which allow physiologically relevant human 3D aligned cardiac microtissues. Moreover, the combination of: i) the low cost, high-content character of the introduced MPS; ii) the possibility of disease modeling and patient-specific screening provided by hiPSC technology; and, iii) the potential to integrate the MPS with other microfluidic based organ systems make our MPS an unique and very powerful tool with a wide range of applications in pharmaceutical industry and fundamental biological investigations.

## Methods

### Differentiation of hiPSC into cardiomyocytes

To generate consistently over 90% cardiomyocytes (CMs), we optimized a previously reported directed cardiac differentiation method^20^, as summarized in Supplementary Figure 1. hiPSCs were cultured on Matrigel-coated 6 well-plates in E8 medium. When the cell confluence reached 80–90%, which is referred as day 0, the medium was switched to RPMI 1640 medium (Life Technologies) containing B27 minus insulin supplement (Life Technologies) and 10 μM CHIR99021 WNT inhibitor (Stemgent). At day 1, the medium was changed to RPMI 1640 medium containing B27 minus insulin supplement. At day 2, medium was replaced to RPMI 1640 medium, B27 supplement without insulin, and 3 μg/mL IWP4 (Stemgent) for 3 days without medium change. On day 5, medium was replaced to RPMI 1640 medium containing B27 minus insulin supplement. On day 7, medium was replaced to a serum-free medium - RPMI 1640 containing B27 with insulin supplement. After day 7, the medium was changed every 3 days. Confluent contracting sheets of beating cells appear at day 8. Flow cytometry on day 20 reveals that the cTnT positive cells account for over 90% of the cell population.

### Immunofluorescence microscopy

One week after loading, the microtissue was fixed in a 4% solution of paraformaldehyde (Santa Cruz Biotechnology) for 15 min, permeabilized with 0.1% Triton-X-100 (Fisher Scientific) for 15 min. After blocking with 3% BSA for 1 h, the microtissue was incubated overnight at 4°C with a primary antibody for sarcomeric alpha actinin (1:100, Sigma-Aldrich). Subsequently, the microtissue was stained with Alexa Fluor-conjugated secondary antibodies (1:100, Life Technologies) overnight at 4°C and with a nuclei-stain applied (DAPI, 4,6-diamidino- 2-phenylindole; 1:500, Sigma-Aldrich). In between each step the device was flushed with PBS at least three times. Confocal microscopy images were acquired using a Zeiss LSM710 laser-scanning microscope (Carl Zeiss, Jena, Germany). Samples were imaged with a 40× planapochromatic NA 1.4 objective.

### Flow cytometry

The efficiency of cardiac differentiation was evaluated using flow cytometry. At day 15–20 of the differentiation, cells were washed with PBS, dissociated using 0.25% trypsin for 10 minutes at 37°C, and then fixed in 4% PFA for 10 minutes at room temperature. Subsequent to two washing steps in FACS buffer solution containing 0.5% w/v of Saponin (Sigma-Aldrich), 5% FBS, 1% BSA (Sigma-Aldrich) in 1× PBS, the cells were incubated for 45 minutes with primary antibodies in FACS buffer solution w/saponin, washed twice in FACS buffer w/saponin, and finally incubated for 45 minutes with secondary antibodies. For flow analysis, cells were washed 2× in FACS buffer w/saponin and resuspended in 1× PBS. All experiments included the appropriate iso-type controls. At least three independent experiments were performed. The following primary and secondary antibodies were used: anti-CD90 Thy1 (mouse monoclonal, 1:250, abcam), Anti-Troponin T Cardiac isoform Ab-1 (mouse monoclonal, 1:100, thermo), Anti-CD31 (rabbit monoclonal, 1:100, abcam), Anti-Calponin (rabbit monoclonal, 1:200, abcam), and Alexa488 (goat-anti-mouse or goat - anti - rabbit, 1:200, Invitrogen). Analysis was performed on the LSR Fortessa Analyzer in the flow cytometry facility at UC Berkeley.

### Semi quantitative PCR

Total RNA was isolated using TRIzol Reagent (Ivitrogen) from undifferentiated hiPSCs as well as from hiPSC-CMs at day 25 of differentiation. DNase I treated total RNA (500 ng) was reverse-transcribed using Superscript II RTase and random hexamers (Gibco). cDNA was diluted 1:4 with sterile distilled water and 5 μL were amplified using PCR Reaction Mix (Sigma). Negative controls were generated in RT reactions in which all reaction components were included except RTase. Reactions were terminated at the exponential phase of amplification and products were analyzed by agarose gel electrophoresis. GAPDH was used for normalization of expression levels of individual genes.

### hiPSC-CMs electrophysiological measurements

Electrophysiology was assessed with a MEA system (MED64, Alpha MED Scientific Inc.). Beating CMs on Day 15 were dissociated using a singularization protocol and replated with a density of 1 million cells/mL on an MEA chip. Prior to measurements the cells were cultured for 7 days using RPMI/B27-C. Throughout the recording period the cells were maintained at 37°C in a sterile environment. Field potential duration of beating CMs were then recorded.

### Device fabrication

The microfluidic devices were fabricated via a two-step photolithography process as described in Supplementary Figure 2. In the first step, the “endothelial-like” barriers and the weir gap were patterned. Thereto, a 2 μm thick layer of SU-8 2001 photoresist (MichroChem Corp, Newton, MA) was spin coated onto silicon wafers (University Wafer, Boston, MA) and exposed to 105 mJ/cm^2^ of UV light using a mask aligner (Karl Suss MA6) for 10 s. Wafers were developed in SU-8 developer (MichroChem Corp, Newton, MA) for 80 s, rinsed in isopropanol, and blow dried with N_2_. In the second step, the media and cell culture channels were fabricated by spinning a 35 μm thick SU-8 3025 (MichroChem Corp, Newton, MA) layer onto the first layer and exposing it to 250 mJ/cm^2^, followed by a 5 min development step. The patterned wafers were baked, and coated with trichloro-1H, 1H, 2H, 2H-perfluorooctylsilane (FOTS, Gelest, PA, USA) using molecular vapor deposition system (AMST MVD100). The masks for both steps were designed in AutoCAD LT (Autodesk Inc., San Rafael, CA) and fabricated on a Micronic MP80+ direct write pattern generator (Micronic Laser Systems, Sweden). PDMS devices were then fabricated. To replica mold the microfluidic devices, uncured polydimethylsiloxane (PDMS, Sylgard 184) was prepared with 1:10 w/w ratio of curing agent to prepolymer, poured onto the wafer and cured overnight at 60°C. After peeling the mold from the wafers, access holes were punched in the PDMS with a 1 mm biopsy punch (Ted Pella). Finally, PDMS devices and microscope glass slides were bonded after exposure to oxygen plasma (Plasma Equipment Technical Services, Livermore, CA) at 60 W for 20 s. To stabilize bonding, the devices were subsequently baked at 80°C for 3 h.

### Device loading

Beating CMs were dissociated using a singularization protocol, as previously described^30^. Briefly, the cells were incubated for 1.5 h in dissociation buffer [1 mg/mL collagenase II (Worthington) with 40 Unit/mL DNase I (BioLabs Inc.) in Hank's balanced salt solution (HBSS)] (Life Technologies) followed by 0.25% trypsin/EDTA (Life Technologies) treatment for 5 min. The cells were resuspended in EB20 media (Knockout DMEM supplemented with 20% fetal bovine serum (FBS), 2 mM l-glutamine, 1× MEM non-essential amino acids (MEM-NEAA), 400 nM 2-mercaptoethanol) (Life Technologies) with 10 μM Y-27632. The devices were hydrophilized and sterilized for 3 minutes at 180 W using O_2_ plasma (PETS Reactive Ion Etcher) and subsequently incubated for 1 h with 20 μg/mL fibronectin (Invitrogen) in PBS (Corning). For cell loading a solution of 4–5 million cells/mL was prepared. 100–200 μL of cell solution was applied to the cell inlet ports of the devices. To achieve a high cell density inside the inlet ports, the devices were stored for 15 min in the incubator to allow for sedimentation of cells. Subsequently, a negative pressure was applied to the cell outlet and media ports using a PhD Ultra syringe pump (Harvard Apparatus) and a flow rate of 10–20 μL/min, leading to a densely packed cell pellet in the cell chamber (Supplementary Movie 2). Following the actual loading step, the devices were stored for 30 min in the incubator to allow cell attachment and tissue formation before starting the flow of media through the media channels. After 24 hours, the media was switched to EB20 (w/o Y-27632). Once the microtissue started beating, the media was switched to a serum-free media (RPMI 1640 containing B27 with insulin supplement). Prior to any experiment, the microtissue was cultured in serum-free media for at least 2–3 days. Throughout the culture time a gravitational flow with an average flow rate of approx. 20 μL/h was used.

### Cardiomyocyte beating analysis

The beating of the micro tissue was characterized using a custom-made motion tracking software analyzing bright field microscope movies as described previously^21^. Briefly, images were partitioned into an array of macroblocks and the motion of each block for subsequent frames determined via an exhaustive search block matching algorithm. Thereby, a corresponding array of motion vectors was obtained for every frame. By averaging the vector amplitude over specific regions of interest, motion velocity tracings are gained, featuring two subsequent characteristic (contraction and relaxation) peaks and allowing for the calculation of parameters such as beat rate and peak velocities. Additionally, the vector characteristics enable a quantification of the degree of alignment of the beating motion.

### Drug testing on the device

All drug testing experiments were performed at least 5 days after loading of the devices whereby cells were loaded at day 15–20 of differentiation. Drugs were dissolved in a serum-free media and serially diluted in the same media. Each concentration of the drug to be tested was preheated for 15–20 min in a water bath at 37°C and subsequently injected in the device. After 30 min, the drug's response on the microtissue was recorded using a Nikon Eclipse TE300 microscope fitted with a QImaging camera. Images were acquired at 10–20 frames per second. A schematic of the experimental protocol used is shown in Supplementary Fig. 3.

### Diffusion characterization

FRAP experiments were performed on fixed tissue samples in the device. An inverted ZEISS LSM 710 confocal microscope, with an argon-ion laser (488 nm), and a 40×, 1.1 NA water objective was used. The experimental setting were: frame size 512 × 256 pixels, zoom factor of 3, circular region of interest 10 μm in diameter, pinhole setting 1 a.u. A solution of 4 kDa FITC–dextran probes (Sigma-Aldrich) were prepared at a concentration of 3.3 mg/mL in PBS. The solution was flowed in the microfluidic device at 20 μL/hr. Experiments started with 10 pre-bleach scanned images at low laser intensity, bleached at scan speed of 12.6 μs at double zoom at 100% laser intensity, and followed by fluorescence recovery at low intensity. Data was normalized by the prebleach fluorescence intensity. All experiments were performed at room temperature.

### Oxygen transport simulations and calculation of non-dimensional mumbers

Oxygen (O_2_) concentration, simulated using COMSOL Multiphysics® (ver. 3.5a) was calculated to reach 0.19 mM within the chamber (Supplementary Fig. 4), slightly lower than the physiological O_2_ concentration of 0.22 mM in blood^31^. O_2_ diffusion was assumed to occur in two ways: a) vertically and laterally through the PDMS, and b) from media in the nutrient channel. Diffusion coefficients of O_2_ in PDMS and culture media were 3.25e-9 and 3.0e-9 m^2^/sec, respectively^32^,^33^. Around 2,000 cells in the cell culture chamber were assumed to consume O_2_ at the rate of 6e-15 mol/min/cell^34^. Velocity and pressure profiles were also modeled in the COMSOL Multiphysics® laminar low module. The governing equation used was the Navier-Stokes Equation. The fluid was assumed to be uniform, Newtonian, and incompressible, with a dynamic viscosity of 0.692 mPa*s and a density of 1.00 g/mL. Volumetric flow at the inlet was set to be 20 μL/hr, and an outlet pressure of 0 Pa was applied. The flow was steady-state and fully developed, with a no-slip condition applying at all device walls. A fine, physics defined mesh was created, and finite element analysis was run to determine fluid pressure and velocity at all points in the device. The geometry of the cell channel and the media channel were rectangular. Peclet number (Pe) and fluidic resistance (R) and was calculated using equations 1 and 2, where u is average flow speed, l is characteristic length, D is diffusion coefficient, η is viscosity.
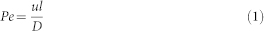

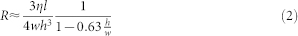


### Statistical analysis

Results indicated as mean ± SD derived from at least three independent experiments. *P* values for the purpose of group comparisons were calculated using Student's *t* test. Differences where *P* <0.05 were regarded as significant.

## Author Contributions

K.E.H., A.M., L.P.L. and P.L. designed the device. A.M. fabricated the device. A.M. and P.L. performed and analyzed the experiments. K.S. and N.M. differentiated the cardiomyocytes. A.M., K.S. and N.M. characterized the cardiomyocytes. S.G.M. and S.H. performed simulations. M.M. made the GCaMP cell line. N.H. and B.R.C. gave biological advice. A.M., P.L. and K.E.H. wrote the manuscript. L.P.L. and K.E.H. designed the study.

## Supplementary Material

Supplementary Information FRAP experiment using 4 kDa FITC-Dextran in the MPS.

Supplementary Information Day 15 - 20 hiPSC-CMs loaded into the MPS at low pressure and low stress.

Supplementary Information hiPSC-CMs in the MPS beat spontaneously at physiological beat rates (55 - 80 beats per minute) in serum-free media without any stimulation.

Supplementary Information GCaMP6 reporter cells in the MPS allow visualization of Ca++ transients via optical microscopy.

Supplementary Information Shows spontaneous baseline beating before Isoproterenol exposure.

Supplementary Information Shows increase in beat rate after 30 min exposure to 1 μM Isoproterenol.

Supplementary Information Shows spontaneous baseline beating before Verapamil exposure.

Supplementary Information Shows decrease in beat rate after 90 min exposure to 1 nM Verapamil.

Supplementary Information Supplementary Information

## Figures and Tables

**Table 1:** Cohort of drugs from different classes that have been tested in our cardiac MPS

Class	Drug	Pharmacology	Average ETPC_unbound_	Safety Margin = IC_50_/Average ETPC_unbound_
Ca^2+^ channel & hERG blocker	Verapamil	IC_50_ = 950 nM	53 nM	18
β-adrenergic agonist	Isoproterenol	EC_50_ = 315 nM	1.75 nM	180
β-adrenergic antagonist	Metoprolol	IC_50_ = 244 μM	2.3 μM	106
hERG blocker	E-4031	IC_50_ = 392 nM	1.9 nM	206

**Table 2:** Verapamil's effect on different models and the cardiac MPS, to calculate the safety margin mean ETPC*_unbound_* of 53 nM^28^ was used

Model	IC_50_ (nM)	IC_50_/Avg. ETPC_unbound_
30 d EBs	103.2 ± 6.03^24^	2
82 d EBs	410.65 ± 40.8^24^	8
2D hiPSCs	169 ± 24^24^	3
Animal (pig ventricular myocyte)	600^36^	11
Animal (pig papillary muscle)	800^36^	15
Cardiac MPS	950	18
