# Supplementary material for: Human iPSC-based Cardiac Microphysiological System For Drug Screening Applications
Source: Sci Rep. 2015 Mar 9;5:8883. doi: 10.1038/srep08883 (PMC4352848; doi:10.1038/srep08883)
Supplement: Supplementary Information [file srep08883-s9.pdf]

Supplementary Information

Human iPSC-based Cardiac Microphysiological System For Drug Screening Applications

Authors: Anurag Mathur[1, 2], Peter Loskill[1, 2], Kaifeng Shao[1], Nathaniel Huebsch[4, 5], SoonGweon Hong[1], Sivan G Marcus[1], Natalie Marks[1], Mohammad Mandegar[4, 5], Bruce R Conklin[4, 5], Luke P Lee[1, 3], and Kevin E Healy[1, 2*]

Affiliations
[1] Department of Bioengineering and California Institute for Quantitative Biosciences (QB3), University of California at Berkeley, Berkeley, California 94720, USA
[2] Department of Materials Science and Engineering, University of California at Berkeley, Berkeley, California 94720, USA
[3] Department of Electrical Engineering and Computer Science, University of California at Berkeley, Berkeley, California 94720, USA
[4]Gladstone Institute of Cardiovascular Disease, San Francisco, California 94158, USA
[5] Department of Medicine, Division of Genomic Medicine, UCSF, San Francisco, California 94143, USA

[*] **Corresponding author**
Kevin E. Healy
370 Hearst Memorial Mining Bldg. #1760,
Berkeley, CA 94720-1760, USA.
Tel.: +1 510 643 3559;
Fax: +1 510 643 5792.
Email: kehealy@berkeley.edu

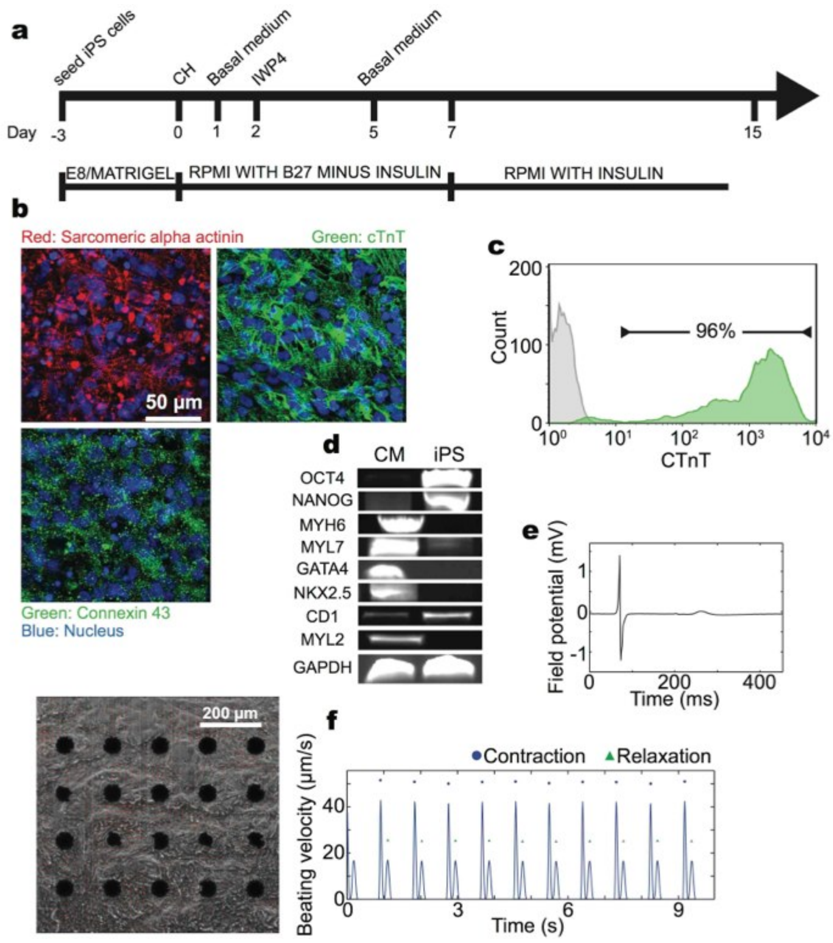

Supplementary Figure 1: Characterization of differentiation of hiPSC into CMs. (a) Schematic representation of the modified differentiation process using small molecules. (b) Confocal images of differentiated CMs expressing cardiac proteins - sarcomeric alpha actinin, cardiac troponin, connexin. (c) Differentiation efficiency of over 90% was confirmed using flow cytometry. (c) Gene expression analysis of ion channel and pluripotency related genes. (e,f) Differentiated CMs show spontaneous beating after day 15. Beating hiPSC-CMs on multielectrode array chip with (e) corresponding field potential measurements and (f) Motion tracings with well-defined contraction and relaxation peaks. Red arrows indicate motion vectors of the beating cells.

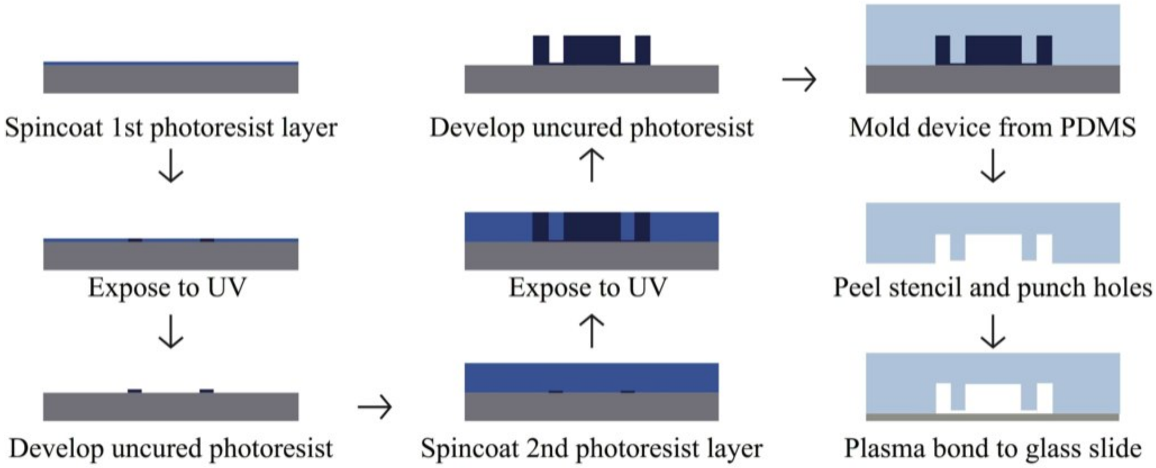

Supplementary Figure 2: MPS fabrication process schematic. The cardiac MPS was fabricated via a two-step photolithography process. In the first step, the “endothelial-like” barriers and the weir gap were patterned, and in the second step, the media and cell culture channels were fabricated. PDMS molds were made from the master wafer and access holes were punched in the PDMS. Finally, PDMS devices and microscope glass slides were bonded.

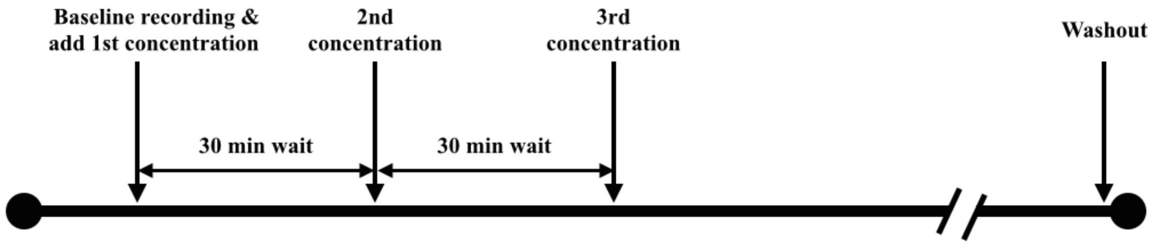

Supplementary Figure 3: Schematic of the drug testing protocol

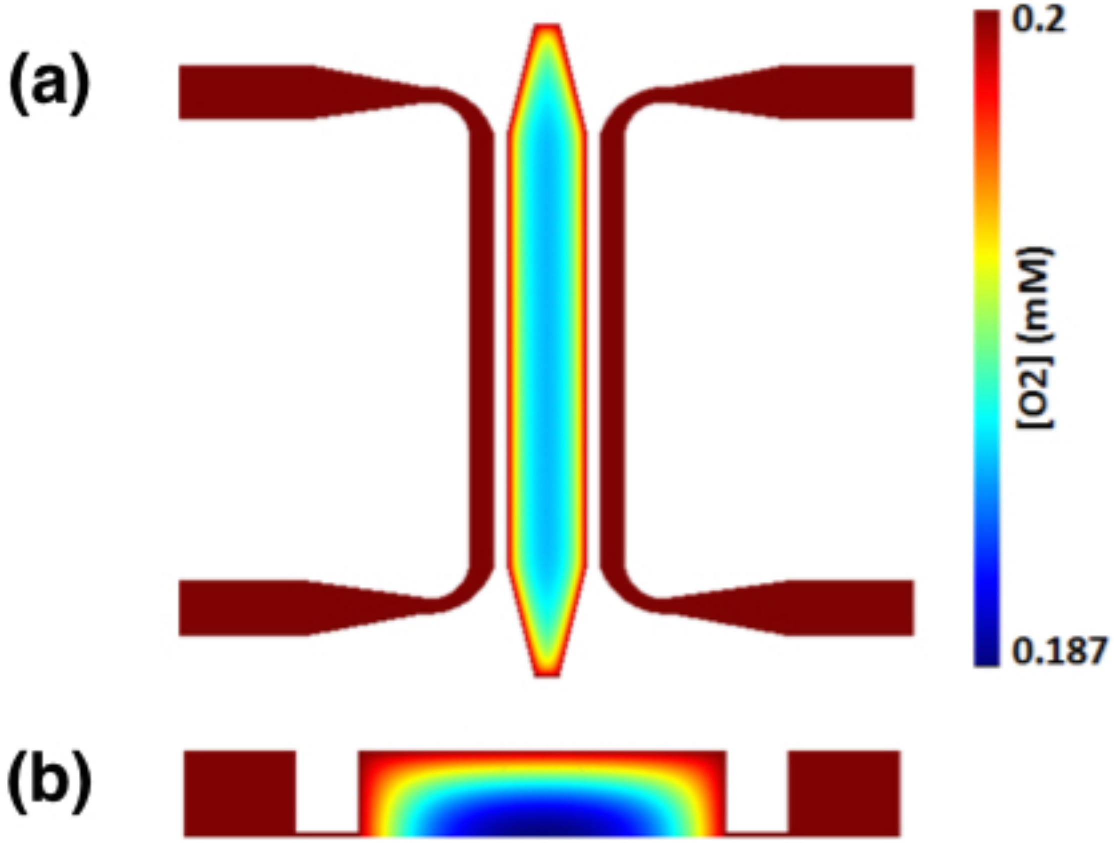

Supplementary Figure 4: Simulation of oxygen diffusion through the MPS revealing a sufficient O_2 concentration (0.19 mM) in the system slightly below the physiological concentration (0.22 mM) in blood.[31] (a) Top view. (b) Cross section. O_2 diffusion was assumed to occur vertically and laterally through the PDMS, and from media in the nutrient channel.

Figure Legends for Supplementary Movies:

Supplementary Movie 1. FRAP experiment using 4 kDa FITC-Dextran in the MPS.

Supplementary Movie 2. Day 15 – 20 hiPSC-CMs loaded into the MPS at low pressure and low stress.

Supplementary Movie 3. hiPSC-CMs in the MPS beat spontaneously at physiological beat rates (55 – 80 beats per minute) in serum-free media without any stimulation.

Supplementary Movie 4. GCaMP6 reporter cells in the MPS allow visualization of Ca^{++} transients via optical microscopy.

Supplementary Movie 5. Shows spontaneous baseline beating before Isoproterenol exposure.

Supplementary Movie 6. Shows increase in beat rate after 30 min exposure to 1 μM Isoproterenol.

Supplementary Movie 7. Shows spontaneous baseline beating before Verapamil exposure.

Supplementary Movie 8. Shows decrease in beat rate after 90 min exposure to 1 nM Verapamil.
